# Internal dosimetry estimates using voxelized reference phantoms for thyroid agents

**DOI:** 10.1093/jrr/rrt125

**Published:** 2013-11-11

**Authors:** E. Hoseinian-Azghadi, L. Rafat-Motavalli, H. Miri-Hakimabad

**Affiliations:** Physics Department, School of Sciences, Ferdowsi University of Mashhad, Vakilabad Boulevard, Mashhad, 91775-1436, Iran

**Keywords:** internal dosimetry, thyroid agents, voxel phantoms, Monte-Carlo method

## Abstract

This work presents internal dosimetry estimates for diagnostic procedures performed for thyroid disorders by relevant radiopharmaceuticals. The organ doses for ^131^Iodine, ^123^Iodine and ^99m^Tc incorporated into the body were calculated for the International Commission on Radiological Protection (ICRP) reference voxel phantoms using the Monte Carlo transport method. A comparison between different thyroid uptakes of iodine in the range of 0–55% was made, and the effect of various techniques for administration of ^99m^Tc on organ doses was studied. To investigate the necessity of calculating organ dose from all source regions, the major source organ and its contribution to total dose were specified for each target organ. Moreover, we compared effective dose in ICRP voxel phantoms with that in stylized phantoms. In our method, we directly calculated the organ dose without using the *S* values or SAFs, as is commonly done. Hence, a distribution of the absorbed dose to entire tissues was obtained. The chord length distributions (CLDs) were also computed for the selected source–target pairs to make comparison across the genders. The results showed that the *S* values for radionuclides in the thyroid are not sufficient for calculating the organ doses, especially for ^123^I and ^99m^Tc. The thyroid and its neighboring organs receive a greater dose as thyroid uptake increases. Our comparisons also revealed an underestimation of organ doses reported for the stylized phantoms compared with the values based on the ICRP voxel phantoms in the uptake range of 5–55%, and an overestimation of absorbed dose by up to 2-fold for Iodine administration using blocking agent and for ^99m^Tc incorporation.

## INTRODUCTION

Thyroid nodules are a common clinical problem, and differentiated thyroid cancer is becoming increasingly prevalent [1]. Therefore, nuclear medicine practitioners recommend and are performing more diagnostic procedures for thyroid diseases using radionuclides ^123^Iodine, ^131^Iodine (sodium iodide) or ^99m^Technetium (pertechnetate). Diagnostic procedures are generally performed for the detection of abnormalities of thyroid anatomy, correlation of anatomy with function, detection of metastatic functioning thyroid tissue, and residual normal tissue after therapy or thyroid uptake measurement [2]. ^99m^Tc is trapped by thyroid cells, while ^131^I and ^123^I is both trapped and organified by them [3]. The amount of dose to entire organs, especially the critical organs such as thyroid, red bone marrow, kidneys or lungs, is the critical issue. However, it is not possible to directly measure the absorbed dose to the organs of the human body from nuclear medicine procedures. Hence, the formalism of the Medical Internal Radiation Dose (MIRD) Committee of the Society of Nuclear Medicine has been long adopted as the accepted calculation method for estimating radiation doses to organs from radionuclides distributed in the body [4].

In MIRD formalism, the time-integrated activity in each source region and the mean absorbed dose to the target organ per unit of nuclear transition are used to derive the absorbed dose to a target organ [5]. The time-integrated activity, though, may be obtained by solving biokinetic models.

Biokinetics describes the behavior of a substance with respect to its uptake, and its transfer to and retention in particular organs and tissues, as well as its subsequent excretion from the body. Biokinetic parameters and models play essential parts in the calculation of radiation doses from incorporated radionuclides. However, for many incorporated radionuclides there are still considerable uncertainties in the biokinetic parameters for humans and thus in the dose estimates [6]. The biokinetic data for a large number of radionuclides were published by the International Commission on Radiological Protection (ICRP) in *ICRP Publication 53* [7], which also reported the organ doses and the effective dose equivalent. These values, which were based on the reference adult, 15-, 10-, 5- and 1-year-old stylized computational phantoms, have contributed significantly to radiation dosimetry in nuclear medicine. Voxel models, constructed from medical image data of real persons, however, give a more realistic description of the human body than the mathematical, stylized phantoms. Thus the ICRP decided to use voxel models to define its reference phantoms to be used for the update of organ dose conversion coefficients [8]. These models (or computational phantoms) represent the reference male and female, and have organ masses in compliance with the reference values, compiled in *ICRP Publication 89* [9]. Some internal dosimetry calculations have been performed recently for voxel phantoms. For example, Lamart *et al.* computed *S* values for ^131^I in the thyroid for the ICRP voxel phantoms [6]. The radionuclides, however, are distributed to other organs as well as to the thyroid, so the *S* values for radionuclides in the thyroid may not sufficient for dose assessment in all target organs. It seems that the effect of other source regions on organ doses could be considerable. So, a more comprehensive computation was performed in this study. Indeed, we specified that the major part of organ dose arises from which source region. Also, we determined the contribution of thyroid as the source region in organ doses. For a more detailed study, the photon and electron contributions were tabulated and the dose distribution in the whole body was visualized in 3D format. Comparison between the two sexes was performed using chord length distribution differences. In addition, effective dose values for the stylized phantom and two voxel phantoms were compared for three radiopharmaceuticals in this paper.

## MATERIALS AND METHODS

### Adult ICRP reference phantoms

In this work, the organ doses were calculated in adult male and female ICRP reference voxel phantoms. Supplementary data files provided by the ICRP represent two 3D matrices of 254 × 127 × 222 and 299 × 137 × 348 dimensions with voxel resolutions of 2.137 × 2.137 × 8 mm^3^ and 1.775 × 1.775 × 4.84 mm^3^ for adult male (AM) and female (AF), respectively. The organ masses and body dimensions of each phantom are reported to match the reference values reported by *ICRP Publication 89* [9]. The densities and elemental compositions for organs and tissues provided in *ICRP Publication 110* [10] were used in the Monte Carlo simulations.

### Estimation of organ doses

The absorbed dose to a target organ *D*(*r*_*T*_) (mGy) is estimated by summing over all source regions (*r*_*S*_), the products of the cumulated activity $\tilde A\left( {r_S } \right)$ (Bq.s) in each source region with the corresponding *S* value. The *S* value in turn, is defined as the mean absorbed dose to the target organ (*r*_*T*_) per unit of nuclear transition of the relevant radionuclide in the source region considered [5, 6]:

(1) $$D\left( {r_T } \right){\rm \; }=\mathop \sum \limits_{r_S } S\left( {r_T \leftarrow r_S } \right)\tilde A\left( {r_S } \right) $$

The cumulated activity $\tilde A\left( {r_S } \right)$ in the source organ/tissue is the time integration of administered activity present within the source region at time *t*.

(2) $$\left( {r_S } \right)=\mathop \int \limits_0^\infty A\left( {r_S, t} \right)dt$$

These cumulated activities for each source region are provided by biokinetic data, which quantify the distribution of radioactivity from the incorporated radionuclide within the body. *ICRP Publication 53* [7] gave biokinetic data from administration of a large number of radiopharmaceuticals, including the three in question. The biokinetic model presented here is a general first-order kinetic model. The activity in an organ or tissue *A*(*r_s_t*) can be described by a multicomponent exponential function:

(3) $$A\left( {r_s\hbox{,} t} \right) = F_s \sum\nolimits_{i = 1}^n {a_i e^{ - \left( {\lambda _i + \lambda _p } \right)t} } $$

where *F*_*S*_ is the fractional distribution to the source organ, i.e. the fraction of the administered substance that would arrive in source organ, over all time, if there were no radioactive decay; and *a*_*i*_ is the fraction of *F*_*S*_ eliminated (taken up) with a biological half-life *T*_*i*_. The biological half-life *T*_*i*_ is associated with λ_*i,*_ which is the biological elimination or uptake constant of the exponential component *I*, and λ_*p,*_ is the physical elimination constant [7].

Another dosimetric quantity of interest is the effective dose E (mSv), which was calculated by the recommended tissue weighting factors *w*_*T*_, allowing for the variations in radiation sensitivity of different organs and tissues to the induction of stochastic effects,

(4) $$E=\mathop \sum \limits_{T} w_T H_T,$$

where *H*_*T*_ is the equivalent dose [8]. Since only photons and electrons are included in the simulations with radiation weighting factor *w*_*R*_ = 1, the corresponding equivalent dose $H_T=\mathop \sum \nolimits w_R D_{T,R} $ is equal to the absorbed dose *D*_*T*_.

### Monte Carlo calculations

In this study, the organ doses were directly calculated from the Monte Carlo simulations, which were carried out separately for photons and electrons. A general purpose Monte Carlo code, MCNPX version 2.4.0 [11] was employed to calculate the absorbed dose for the ICRP voxel phantoms. The male and female phantoms were incorporated into the MCNPX lattice file. Organ- and tissue-specific density and elemental composition were implemented into the material card of the MCNPX code.

The whole body tissues were defined as source regions simultaneously in the SDEF card. However the source probability (*sp*) was so determined to account for different uptakes of source regions:

(5) $${\rm source}\,{\rm probability =} \ \widetilde{{\rm A}}_{\rm S} \cdot \displaystyle{{{\rm organ}\,{\rm volume}} \over {{\rm total}\,{\rm source}\,{\rm organ}\,{\rm volume}}}. $$

Indeed, thyroid, stomach (stomach wall and contents), small intestine (small intestine wall and contents), kidneys, and bladder contents are the main source organs for incorporation of radioactive iodine. Remaining tissues, however, take up iodine as a uniform source of much weaker intensity. As an example, *sp* values for 15% uptake of ^131^I are 36.48, 1.5 and 0.18 for thyroid, urinary bladder contents and liver, respectively. Gastrointestinal contents, except for stomach and SI contents, were not included in the remaining tissues as a source. It should be noted that the radioactive source was described as a uniform source of radiation within any source region. In fact, this method was implied to obtain organ doses directly from the Monte Carlo simulations.

The simulations provided the dose (MeV/g), i.e. energy deposition (MeV) per unit mass (g), in each target organ (*T*) per emitted particle. The dose per particle was multiplied by the total photon or electron yield per decay and summed to obtain the absorbed dose (mGy/MBq). The dose was scored using track length estimate of heating tally (F6) for photons (kerma approximation) and energy deposition (+F6) for electrons. The mode P and P E was selected for photons and electrons, respectively. Furthermore, to make comparisons between the *S* values in this study and Lamart *et al*. [6], the energy deposition in the target organs was scored using both the F6 and * F8 tally.

To better understand the main source of absorbed dose to any target organ, all source regions with their corresponding weighting factors were defined separately: thyroid, stomach, small intestine, kidneys, bladder contents and remaining tissues. To compute the fraction of absorbed dose caused by any source region, the organ dose caused by separate source regions was multiplied by the corresponding cumulated activity and divided by the total absorbed dose.

The spectra published in ENSDF decay data [12] with yields >0.1% were employed for the estimation of the organ doses. The beta spectrum of ^131^I was approximated using the Fermi function with respect to the maximum beta energy. Auger electrons were determined by their average energies, and conversion electrons were determined by their maximum energy for subshell. Absorbed doses to the active red marrow and the endosteal region (bone surface) in both ICRP voxel phantoms were estimated by using methods published in *ICRP Publication 116* [13].

For a more detailed study, additional MCNPX mesh tallies were used to graphically displaying the dose in voxels for the models. Mesh tally Type 1 with the ‘pedep’ option and Type 3 with the ‘total’ option were used for photon and electron sources, respectively, and score the average energy deposition per unit volume (MeV/cm^3^/source-particle). The visualization was performed in two arrays of 254 × 222 and 299 × 348 dimensions, crossing mid-depth in the male and female phantoms, respectively. A rectangular grid was defined, overlaid exactly on the lattice geometry. Therefore, the energy deposited per unit volume in each voxel could be converted to energy per unit mass by dividing by each voxel's density. The energy deposited per unit mass (MeV/g/source-particle) in each voxel then converted to absorbed dose (mGy/MBq) for visualization on the dose distribution map.

### Calculation of chord length distributions

The dose delivered to a target organ from a source region is mainly governed by the activity in the source and the attenuation between the source and target, the latter being a function of the distance between the two organs and the density and elemental composition of the intervening tissue [6]. To explain the differences in organ doses, we computed chord length distributions (CLDs).

A CLD is a relative number histogram of distances between the points in the source and target regions. To specify the position of each point, first, the voxel position was derived from a random sampling of the voxel indices, which belong to source and target regions respectively; then, a point in each voxel was selected. The CLDs were generated by randomly sampling 1 million points in each source organ and each target organ, and assessing the distances between pairs.

### RESULTS

**Fig. 1.**
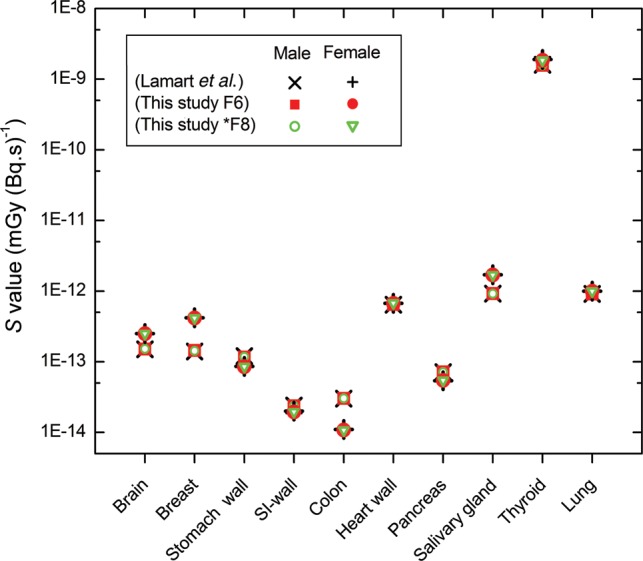
Comparison of *S* values for adult male and female ICRP reference phantoms scored using F6 and *F8 tally in this study and from Lamart *et al.* [6].

The process of the present calculations was validated by comparing the *S* values calculated from adult male/female ICRP reference phantoms, with the *S* values for ^131^I from Lamart *et al.* [6]. Figure 1 indicates that the *S* values calculated in this study (using F6 and *F8 tally) were in good agreement with those from Lamart *et al.* [6]. The results for each radiopharmaceutical come in the following sections. We calculated the absorbed dose for the total of target organs considered in *ICRP Publication 110* [10], as listed in Tables 1–3.

**Table 1.** Organ doses (mGy/MBq) in addition to photon, thyroid and major source organ contributions for 15% thyroid uptake of ^131^I Iodide in male and female phantoms

^131^I Sodium Iodide	AM					AF				
Target Organ	Total	Photon %	Thyroid %	Major Source Organ %		Total	Photon %	Thyroid %	Major Source Organ %	
Active red marrow	1.09E−01	90	72	—		1.27E−01	89	72	—	
Colon	4.85E−02	73	8	25	Self-dose	4.92E−02	70	3	30	Self-dose
Lungs	1.67E−01	82	71	—		1.84E−01	80	71	—	
Stomach wall	5.56E−01	19	3	94	St wall + cont.	5.92E−01	18	2	95	St wall + cont.
Breasts	4.41E−02	70	42	—		8.15E−02	81	67	—	
Testes/Ovaries	2.33E−02	50	0	55	Self-dose	6.01E−02	75	1	36	Bladder cont.
Urinary bladder wall	1.40E−01	70	0	82	Bladder cont.	1.57E−01	65	0	78	Bladder cont.
Esophagus	1.07	87	97	—		1.209716	92	97	—	
Liver	5.33E−02	77	35	—		5.98E−02	75	32	—	
Thyroid	207.19	6	∼100	—		242.90	6	∼100	—	
Endosteal region	5.71E−02	80	60	—		7.11E−02	81	61	—	
Brain	3.91E−02	69	51	—		5.48E−02	74	60	—	
Salivary gland	1.42E−01	91	85	—		2.44E−01	94	91	—	
Skin	3.77E−02	70	51	—		4.64E−02	72	53	—	
Adrenal	5.18E−02	79	25	—		6.25E−02	76	20	24	St wall + cont.
ET	2.10E−01	95	92	—		3.31E−01	95	93	—	
Gall bladder wall	5.08E−02	70	22	—		6.20E−02	74	19	21	St wall + cont.
Heart wall	1.23E−01	90	69	—		1.27E−01	88	70	—	
Kidney	7.27E−02	50	11	57	Self-dose	8.52E−02	52	8	55	Self-dose
LN	3.27E−01	61	87	—		2.21E−01	79	78	—	
Muscle	5.62E−02	77	58	—		7.79E−02	75	63	—	
Oral mucosa	1.15E−01	90	83	—		2.47E−01	94	91	—	
Pancreas	6.77E−02	81	14	27	St wall + cont.	8.24E−02	78	9	34	St wall + cont.
SI-wall	2.32E−01	22	1	92	SI wall + cont.	2.67E−01	24	1	88	SI wall + cont.
Spleen	6.31E−02	80	32	—		7.69E−02	80	21	40	St wall + cont.
Thymus	1.15	95	98	—		1.03	98	97	—	
Prostate/Uterus	6.34E−02	79	0	59	Bladder cont.	8.03E−02	80	0	51	Bladder cont.
Tongue	1.35E−01	91	85	—		2.68E−01	95	91	—	
Tonsils	9.20E−02	84	75	—		1.97E−01	93	89	—	
LN-ET	6.25E−01	97	95	—		6.94E−01	98	96	—	
LN-Th	1.84	79	97	—		3.25	70	99	—	
Eye lenses	3.16E−02	80	64	—		4.99E−02	79	68	—	
Pituitary gland	7.99E−02	60	44	—		9.78E−02	84	75	—	
Spinal cord	2.47E−01	94	89	—		3.18E−01	95	90	—	
Ureters	5.66E−02	74	5	29	SI wall + cont.	6.26E−02	75	4	31	SI wall + cont.
Adipose	6.44E−02	69	56	—		5.99E−02	69	51	—	
Trachea	2.75	95	99	—		3.20	98	99	—	
Bronchi	1.53E−01	90	79	—		1.74E−01	91	81	—	
Gall bladder contents	4.82E−02	72	23	27	Self-dose	5.99E−02	75	20	27	Self-dose
Cartilage	2.05E−01	92	87	—		1.03E−01	77	71	—	
Heart contents	1.05E−01	89	65	—		1.04E−01	86	63	—	
Remainder tissues	2.05E−01	80	73	—		2.23E−01	83	71	—	
**Effective dose (mSv/MBq)**	**8.48**					**9.93**				

The effective dose is also listed in the end of table.

### ^131^I Sodium Iodide

**Fig. 2.**
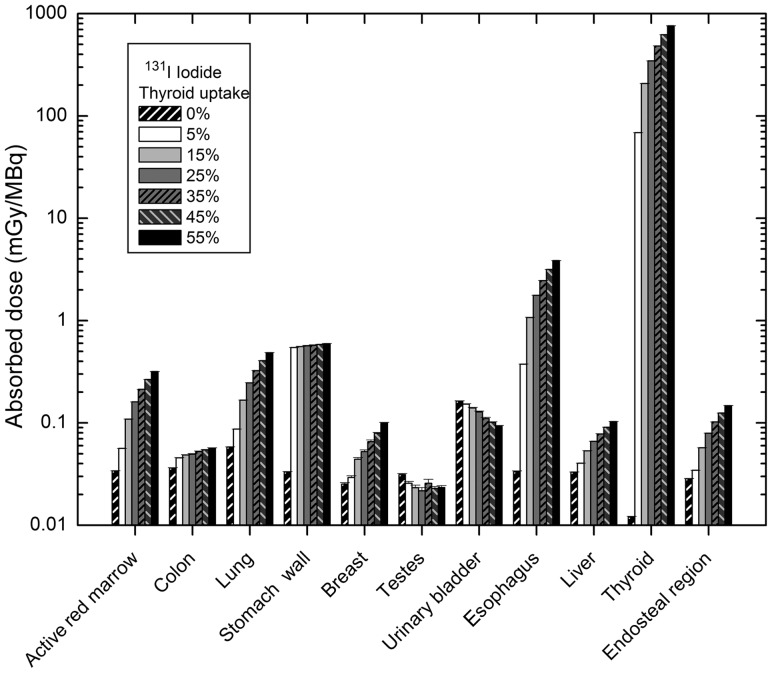
Comparison of absorbed dose to 11 major target organs for different thyroid uptakes of ^131^I in male phantom.

**Fig. 3.**
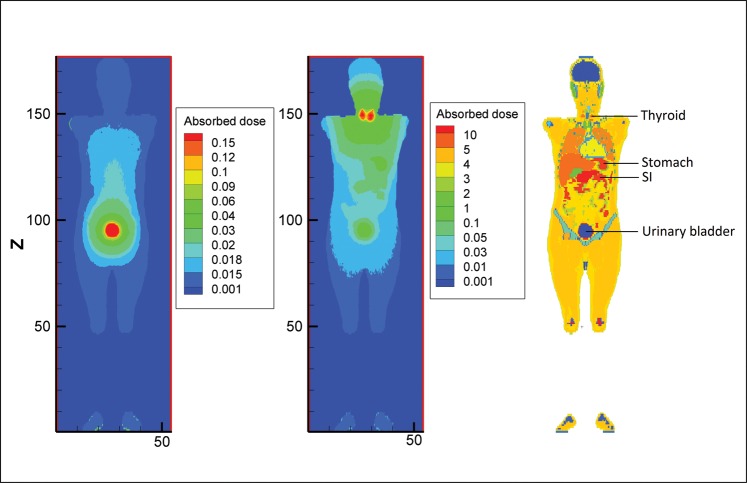
Contour plot of voxel doses at the mid-depth of male model in the case of blocked thyroid and 15% uptake of ^131^I together with MCNP geometry plot of the phantom (from left to right).

The absorbed dose to target organs from ^131^I distributed within the body was calculated at six levels (5, 15, 25, 35, 45 and 55%) of thyroid uptake as well as blocked thyroid (uptake 0%). A total of 10^7^ particles in each program were simulated to give a statistical error of < 1% for most target organs and 5% for mesh tallies. The number of simulated particles was the same for ^123^I and ^99m^Tc. The absorbed doses for 11 major target organs in the adult male phantom are illustrated in Fig. 2. The organs and tissues that were selected for comparison of absorbed dose in various thyroid uptakes were active red marrow, colon, lungs, stomach wall, breasts, gonads, urinary bladder wall, esophagus, liver, thyroid and endosteal region (bone surface). As thyroid uptake varies from 0–55%, the absorbed dose to most organs increases, for instance, the thyroid, esophagus and lungs. However for the colon, stomach wall, and testes, it remains relatively constant, and for the urinary bladder wall, it decreases. To better understand the differences between the absorbed dose distribution for 15% thyroid uptake and blocked thyroid, a 3D plot of the adult male ICRP voxel phantom has been shown in Fig. 3. It should be noted that the blocked thyroid caused a higher dose to the bladder and adjacent organs.

The organ doses from the Monte Carlo calculations using the ICRP voxel phantoms could be compared with those for the ORNL phantom reported in *ICRP Publication 53* [7]. One can readily observe that organ doses calculated in this study for 0–55% uptakes of ^131^I in the thyroid indicated the same ascending and descending trends as seen in the stylized phantom.

**Fig. 4.**
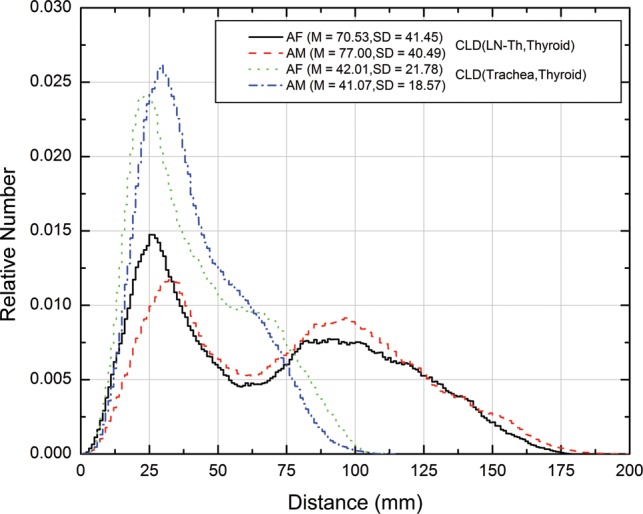
Comparison of CLDs for (LN-Th–thyroid) and (trachea–thyroid) pairs in male and female. M and SD in the legend stand for mean and standard deviation, respectively.

Normal values of fractional thyroid uptake used in the *MIRD Dose Estimate Report No. 5* [14] varied from 0.05–0.25 and were considered to encompass a range appropriate to the adult euthyroid population of the USA. Absorbed doses (mGy/MBq) to target organs for 15% uptake, representative of normal thyroid uptake, are shown in Table 1, in which calculated values for the adult male ICRP voxel phantom are compared with those for the adult female ICRP voxel phantom. Target organs in the female phantom generally receive greater energy depositions than those in the male phantom. As expected, the thyroid gland has the largest absorbed dose, 207.19 and 242.90 mGy/MBq in the male and female, respectively. The second and third greatest absorbed doses (2.75 and 1.84 mGy/MBq) are found in the trachea and the lymphatic nodes of the thoracic airways (LN-Th) in the male phantom, while in the female model, absorbed doses to the LN-Th and the trachea—3.25 and 3.20 mGy/MBq, respectively—are relatively similar. The CLDs for (LN-Th, thyroid) and (trachea, thyroid) pairs in the male and female are shown in Fig. 4.

**Fig. 5.**
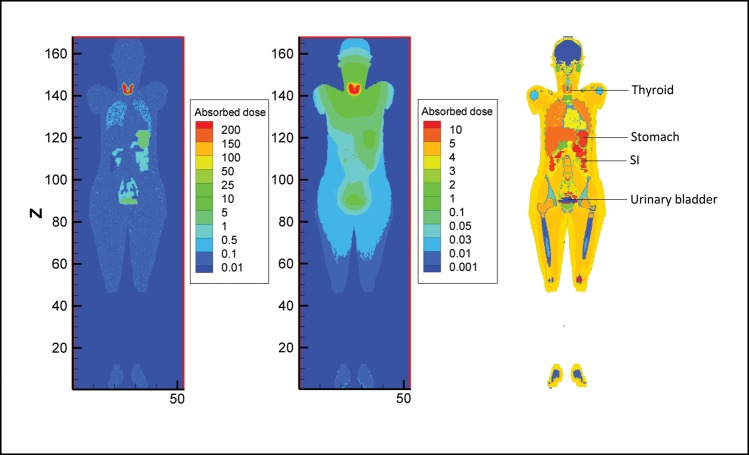
Contour plot of voxel doses at the mid-depth of female model in the case of electron and photon sources for 15% thyroid uptake of ^131^I together with MCNP geometry plot (from left to right).

The second column of Table 1 lists the contribution of photons in organ doses. In all organs except the thyroid, stomach wall and small intestine wall, the contribution of photons is more than that of electrons. For example, in the thyroid gland, 94% of the absorbed dose arises from electrons (beta particles and conversion electrons) for both male and female phantoms. Absorbed doses in voxels at the mid-depth of the phantoms (obtained from mesh tallies) are drawn in a contour plot. Comparison of absorbed doses from photons and electrons of ^131^I is presented in a 3D plot of the female phantom (Fig. 5). The energy deposited from photons is more dispersed around source regions relative to local deposition of the energy from electrons.

The contribution of the thyroid as a source organ in organ doses are tabulated in Table 1. In cases where the thyroid was not the major source organ, the name and contribution of the major source organ has been specified in the next column. A total of 10^8^ particles in each program were simulated to give a statistical error of < 1% for most target organs. The number of simulated particles is the same for ^123^I and ^99m^Tc. The proportion of energy deposition from ^131^I in thyroid is > 50% for most target organs such as lungs, esophagus, salivary glands, and skin. The thyroid receives ∼100% of absorbed dose from ^131^I distributed within the thyroid in both the male and female. In some target organs like breasts, liver, adrenals and spleen, particles emitted from the thyroid contribute < 50% but are still the major source compared with other sources in the body of the male phantom. The colon, stomach wall, testes, small intestine wall, kidneys and gall bladder contents receive most of their absorbed dose from themselves. Another important source organ is the stomach wall and contents, which is responsible for the absorbed dose to the pancreas in both genders, and to the spleen, adrenals and gall bladder wall in the female phantom. Although the thyroid uptake of ^131^I from blood is considerably greater than from all other organs, we have shown that it is not sufficient to assume the thyroid is the only source region.

### ^123^I Sodium Iodide

**Fig. 6.**
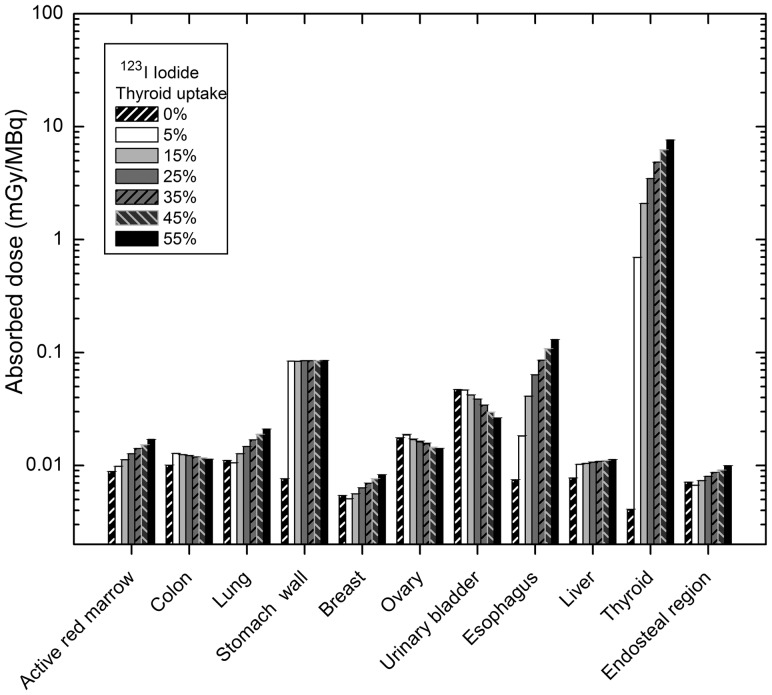
Comparison of absorbed dose to 11 major target organs for different thyroid uptakes of ^123^I in female phantom.

The absorbed dose to target organs from administration of ^123^I was calculated for a thyroid uptake of 0–55%. The absorbed doses for 11 major target organs in the female phantom are illustrated in Fig. 6. As thyroid uptake increases, most organs (e.g. thyroid, esophagus, lungs and active red marrow) receive a larger absorbed dose. However, the incremental trends in some organs (e.g. lungs) is slower compared with those for ^131^I. Moreover for colon, stomach wall and liver, dose with respect to thyroid uptake remains relatively constant, and for urinary bladder wall and ovaries it decreases.

**Fig. 7.**
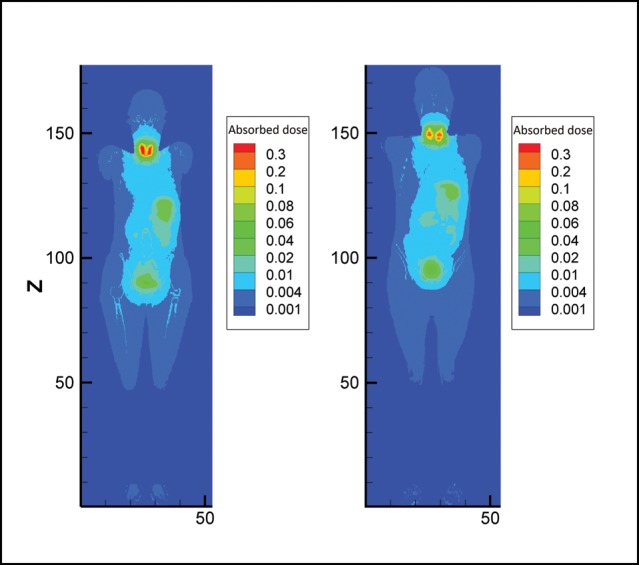
Contour plot of voxel doses at the mid-depth of female and male voxel phantoms in the case of 15% thyroid uptake of ^123^I (from left to right).

The absorbed dose (mGy/MBq) to target organs for 15% uptake is shown in Table 2. As expected, the thyroid gland has the largest absorbed dose: 1.78 and 2.08 mGy/MBq in the male and female phantom, respectively. The second greatest absorbed dose (8.74 × 10^−2^ and 1.05 × 10^−1^ mGy/MBq) is found for the trachea in the male and female phantoms, respectively. The stomach wall had the third greatest absorbed dose: 7.76 × 10^−2^ and 8.36 × 10^−2^ mGy/MBq in the male and female phantoms, respectively. Comparison of organ doses from photons of ^123^I is presented in a 3D plot of the female and male phantoms in Fig. 7. This figure illustrates that target organs in the female phantom generally receive greater energy depositions than those in the male phantom; note the hotter shade of the thyroid region in the 3D plot of the female phantom.

**Table 2.** Organ doses (mGy/MBq), in addition to photon, thyroid and major source organ contributions for 15% thyroid uptake of ^123^I Iodide in male and female phantoms

^123^I Sodium Iodide	AM					AF				
Target Organ	Total	Photon %	Thyroid %	Major Source Organ %		Total	Photon %	Thyroid %	Major Source Organ %	
Active red marrow	9.18E−03	89	20	—		1.12E−02	89	21	—	
Colon	1.22E−02	91	1	32	SI wall + cont.	1.25E−02	90	0	37	SI wall + cont.
Lungs	1.14E−02	76	25	40	Self-dose	1.27E−02	74	25	42	Self-dose
Stomach wall	7.76E−02	46	0	92	St wall + cont.	8.36E−02	47	0	92	St wall + cont.
Breasts	4.62E−03	75	7	31	Self-dose	5.59E−03	76	18	40	Self-dose
Testes/Ovaries	4.67E−03	77	0	33	Self-dose	1.70E−02	93	0	44	Bladder cont.
Urinary bladder wall	4.06E−02	95	0	85	Bladder cont.	4.20E−02	95	0	81	Bladder cont.
Esophagus	3.49E−02	97	79	—		4.10E−02	96	81	—	
Liver	8.72E−03	88	4	31	Self-dose	1.04E−02	88	3	34	St wall + cont.
Thyroid	1.78	24	99	—		2.08	24	100	—	
Endosteal region	5.79E−03	83	14	—		7.33E−03	83	15	—	
Brain	3.72E−03	71	8	70	Self-dose	4.47E−03	71	12	69	Self-dose
Salivary gland	5.50E−03	81	45	—		8.75E−03	86	56	—	
Skin	3.59E−03	72	12	34	Self-dose	4.45E−03	72	13	32	Self-dose
Adrenal	1.07E−02	90	2	29	St wall + cont.	1.30E−02	91	2	41	St wall + cont.
ET	7.93E−03	86	60	—		1.19E−02	87	64	—	
Gall bladder wall	9.06E−03	88	2	22	St wall + cont.	1.32E−02	88	2	31	St wall + cont.
Heart wall	1.18E−02	91	15	39	St wall + cont.	1.11E−02	89	17	30	St wall + cont.
Kidney	1.36E−02	76	1	43	Self-dose	1.70E−02	79	1	38	Self-dose
LN	1.74E−02	93	31	—		1.69E−02	92	25	—	
Muscle	5.58E−03	81	14	46	Self-dose	7.11E−03	82	15	40	Self-dose
Oral mucosa	5.11E−03	78	36	—		8.85E−03	86	55	—	
Pancreas	1.75E−02	94	1	38	St wall + cont.	2.26E−02	94	1	44	St wall + cont.
SI-wall	3.49E−02	53	0	82	SI wall + cont.	4.21E−02	55	0	77	SI wall + cont.
Spleen	1.21E−02	92	3	45	St wall + cont.	1.85E−02	93	2	63	St wall + cont.
Thymus	3.50E−02	97	87	—		3.43E−02	96	83	—	
Prostate/Uterus	1.81E−02	94	0	73	Bladder cont.	2.28E−02	95	0	62	Bladder cont.
Tongue	6.00E−03	79	38	—		9.60E−03	87	55	—	
Tonsils	5.12E−03	73	24	—		7.68E−03	88	46	—	
LN-ET	2.04E−02	93	80	—		2.25E−02	94	79	—	
LN-Th	5.30E−02	97	89	—		8.20E−02	97	93	—	
Eye lenses	1.91E−03	86	16	61	Self-dose	4.24E−03	49	12	29	Self-dose
Pituitary gland	2.94E−03	99	20	45	Brain	4.69E−03	82	27	31	Brain
Spinal cord	9.41E−03	89	48	—		1.15E−02	90	51	—	
Ureters	1.47E−02	95	0	41	SI wall + cont.	1.70E−02	92	0	42	SI wall + cont.
Adipose	6.09E−03	80	11	35	Self-dose	5.91E−03	76	10	47	Self-dose
Trachea	8.74E−02	99	95	—		1.05E−01	99	95	—	
Bronchi	9.33E−03	88	29	—		1.02E−02	87	32	—	
Gall bladder contents	9.17E−03	87	2	22	SI wall + cont.	1.26E−02	89	2	30	St wall + cont.
Cartilage	1.15E−02	91	47	—		8.33E−03	85	22	—	
Heart contents	1.11E−02	90	13	38	St wall + cont.	1.12E−02	88	12	33	St wall + cont.
Remainder tissues	1.54E−02	84	24	—		1.87E−02	85	22	—	
**Effective dose (mSv/MBq)**	**9.01E−02**					**1.05E−01**				

The effective dose is also listed in the end of table.

The energy deposition from photons is more than 50% of that from electrons in most target organs. In all organs except the thyroid and stomach wall in both genders, the contribution of photons is more than that of electrons. For example, in the thyroid gland 76% of the absorbed dose arises from electrons (conversion electrons only) for both male and female phantoms.

The contribution of absorbed dose to target organs such as the thyroid, esophagus, salivary glands, ET airways (anterior and posterior nasal passages down to larynx), thymus, and trachea from ^123^I in the thyroid as the source organ is uppermost. For example, the thyroid receives ∼ 99% and ∼ 100% of the absorbed dose from ^123^I distributed within itself in the male and female, respectively. In most target organs like the lungs, breasts, brain and skin, emitted particles from the thyroid contribute < 50%, and these organs receive most of their absorbed doses from themselves. Another important source organ is the stomach wall and contents, which is responsible for the absorbed dose to the adrenals, heart wall, gall bladder wall, pancreas and spleen in both genders, and to the liver in the female phantom. It is readily seen that the thyroid is not the only major source region.

### Pertechnetate

**Fig. 8.**
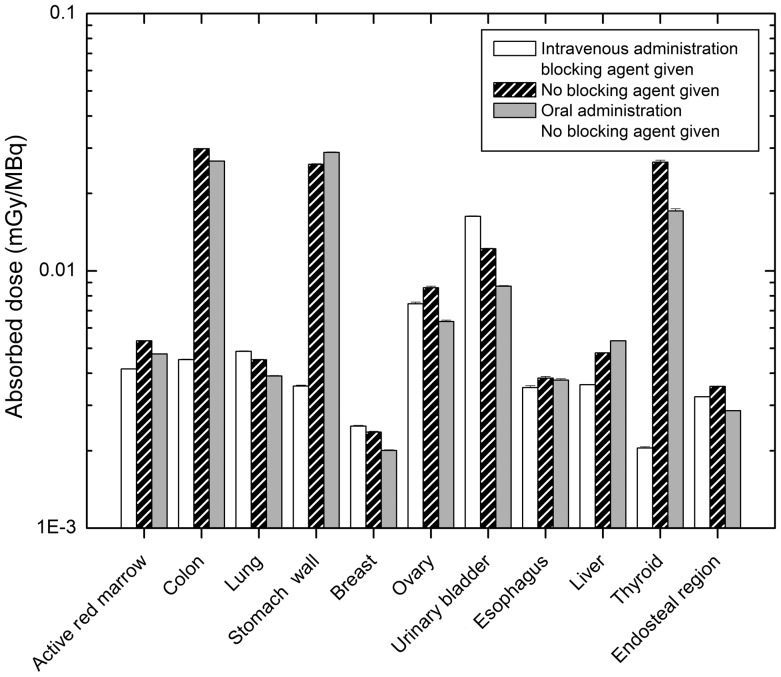
Comparison of absorbed dose to 11 major target organs for different thyroid uptakes of ^99m^Tc in female phantom.

**Fig. 9.**
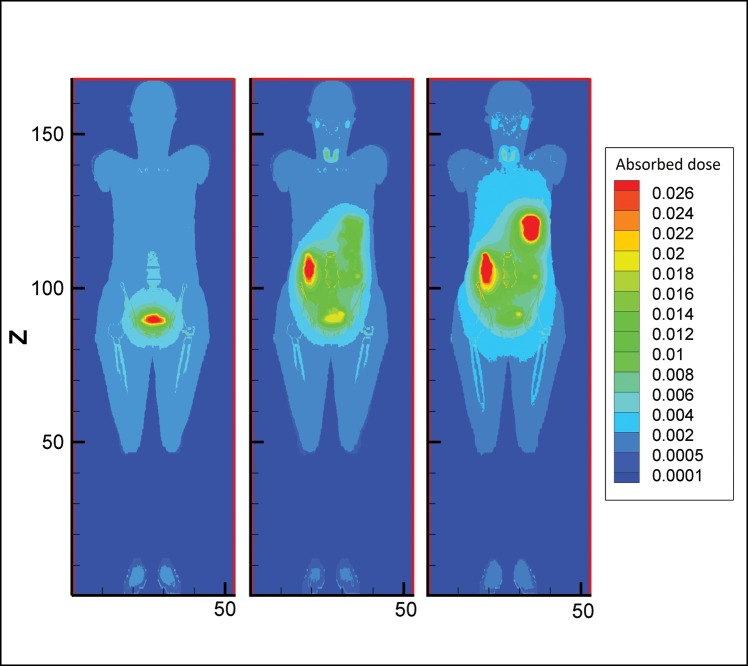
Contour plot of voxel doses at the mid-depth of female model in the case of intravenous administration of ^99m^Tc with Blocking agent, without blocking agent and also when this compound was given orally (from left to right).

Organ dose estimation was performed for intravenous administration of pertechnetate (with or without blocking agent) and also for when this compound given orally. The absorbed doses for 11 major target organs are illustrated in Fig. 8. Most organs such as the thyroid, colon and lungs received a larger absorbed dose when ^99m^Tc was given intravenously than the dose estimated for oral administration. Conversely, the absorbed dose to the stomach wall and some adjacent organs (e.g. pancreas, spleen and liver) was smaller after intravenous administration. Furthermore, when the blocking agent was given, the absorbed dose to the urinary bladder wall and testes was greater. To better understand the differences between these three modes of administration, a 3D plot of absorbed dose in voxels of the female phantom is shown in Fig. 9. As is indicated in this figure, the stomach wall and colon generally receive greater energy depositions when given orally (note the hotter shade of the stomach region in the case of oral incorporation).

Organ doses (mGy/MBq) for intravenous administration are shown in Table 3. The thyroid gland does not have the largest absorbed dose. The greatest absorbed dose (2.72 × 10^−2^ and 2.98 × 10^−2^ mGy/MBq in male and female models, respectively) is found in the colon. The absorbed dose to the thyroid is 2.20 × 10^−2^ and 2.65 × 10^−2^ mGy/MBq in male and female phantoms, respectively. The stomach wall has a greater absorbed dose than the thyroid in the male phantom (2.60 × 10^−2^ mGy/MBq).

The energy deposition from photons is more than 50% of that from electrons in most target organs except for the thyroid, stomach wall and salivary glands in both genders. For example, in the thyroid gland 27% and 26% of the absorbed dose arises from photons for male and female phantoms, respectively.

The main contribution of the absorbed dose to most target organs (e.g. thyroid, esophagus and salivary glands) is from themselves. For example, 92% and 90% of the absorbed dose from ^99m^Tc distributed within the thyroid is self-dose in male and female, respectively. Another important source organ is the right colon wall (the ascending and transverse colon wall), which is responsible for the absorbed dose to the adrenals and spleen in the female phantom. It is readily seen that the thyroid is not the only major source region.

### Comparison of effective dose

**Fig. 10.**
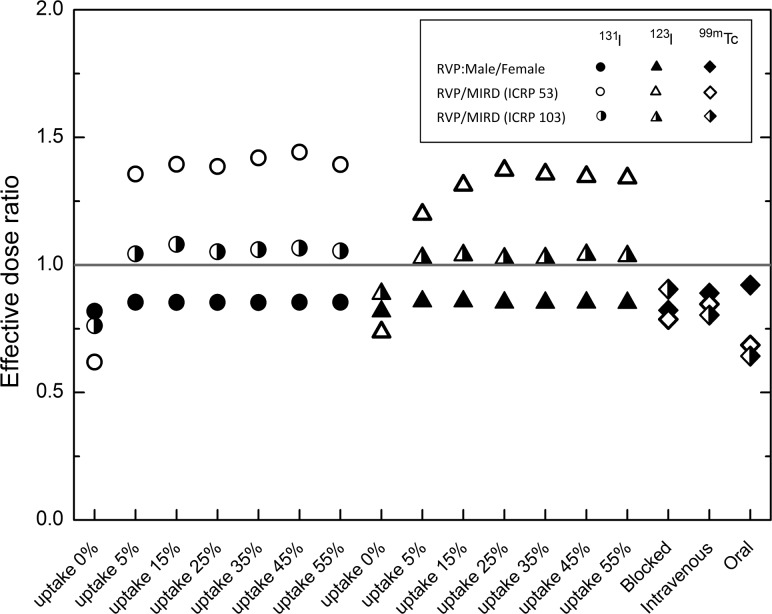
Effective dose ratio for male to female voxel phantoms; the gender-averaged effective dose of this study for the reference voxel phantom (RVP) to the effective dose equivalent for the MIRD phantom (*ICRP Publication 53* method); and the gender-averaged effective dose of this study for the reference voxel phantom (RVP) to the effective dose equivalent for the MIRD phantom (*ICRP Publication 103* method).

‘Effective dose’ is defined for the gender-averaged reference person [8], nevertheless this value has been tabulated for male and female phantoms separately in Tables 1–3. Ratios of the male to female effective dose are plotted for the three radiopharmaceuticals in Fig. 10. In general, organs in the female phantom received greater energy depositions than those in the male phantom. As a result, the effective dose would be higher in the female phantom.

**Table 3.** Organ doses (mGy/MBq), in addition to photon, thyroid and major source organ contributions for intravenous administration of ^99m^Tc pertechnetate in male and female phantoms

^99m^Tc Pertechnetate	AM					AF				
Target Organ	Total	Photon %	Thyroid %	Major Source Organ %		Total	Photon %	Thyroid %	Major Source Organ %	
Active red marrow	4.23E-03	88	1	—		5.35E-03	89	1	—	
Colon	2.72E-02	52	0	62	R Colon wall	2.98E-02	55	0	60	R Colon wall
Lungs	4.31E-03	70	1	49	Self-dose	4.51E-03	66	1	55	Self-dose
Stomach wall	2.60E-02	45	0	72	Self-dose	2.59E-02	41	0	77	Self-dose
Breasts	2.20E-03	78	0	31	Self-dose	2.36E-03	71	1	45	Self-dose
Testes/Ovaries	2.42E-03	77	0	42	Self-dose	8.60E-03	93	0	22	L Colon Cont.
Bladder wall	1.05E-02	94	0	64	Bladder Cont.	1.22E-02	93	0	54	Bladder Cont.
Esophagus	4.09E-03	86	8	16	Self-dose	3.83E-03	85	10	20	Self-dose
Liver	5.70E-03	91	0	23	Self-dose	4.80E-03	87	0	30	Self-dose
Thyroid	2.20E-02	27	92	—	Self-dose	2.65E-02	26	90	—	Self-dose
Endosteal region	2.75E-03	82	0	—		3.55E-03	84	0	—	
Brain	1.81E-03	71	0	69	Self-dose	2.08E-03	70	0	71	Self-dose
Salivary gland	8.42E-03	33	0	88	Self-dose	1.06E-02	33	1	86	Self-dose
Skin	1.74E-03	72	0	33	Self-dose	2.05E-03	72	0	33	Self-dose
Adrenal	5.47E-03	92	0	13	SI Cont.	5.75E-03	88	0	14	St wall
ET	1.82E-03	74	3	33	Self-dose	2.50E-03	76	4	28	Self-dose
Gall bladder wall	8.91E-03	94	0	37	R Colon Cont.	6.11E-03	90	0	14	SI Cont.
Heart wall	4.44E-03	88	1	17	Self-dose	4.06E-03	84	1	22	Self-dose
Kidney	7.09E-03	87	0	22	Self-dose	7.57E-03	86	0	23	Self-dose
LN	5.94E-03	91	1	12	R Colon Cont.	6.84E-03	91	1	15	R Colon wall
Muscle	2.68E-03	81	0	48	Self-dose	3.47E-03	82	0	40	Self-dose
Oral mucosa	2.01E-03	73	1	32	Self-dose	2.38E-03	82	3	30	Self-dose
Pancreas	1.05E-02	95	0	23	R Colon Cont.	9.51E-03	93	0	21	SI Cont.
SI-wall	8.95E-03	94	0	24	SI Cont.	1.15E-02	94	0	22	SI Cont.
Spleen	5.30E-03	91	0	16	Self-dose	5.95E-03	89	0	25	St wall
Thymus	2.92E-03	82	12	33	Self-dose	3.14E-03	82	11	25	Self-dose
Prostate/Uterus	6.10E-03	92	0	48	Bladder Cont.	9.92E-03	93	0	32	Bladder Cont.
Tongue	2.13E-03	75	2	50	Self-dose	2.74E-03	77	3	32	Self-dose
Tonsils	2.19E-03	73	1	36	Self-dose	2.46E-03	83	2	30	Self-dose
LN-ET	2.32E-03	80	9	24	Self-dose	3.39E-03	71	7	19	Salivary glands
LN-Th	3.57E-03	82	14	16	Self-dose	3.94E-03	82	20	—	
Eye lenses	1.26E-03	68	0	38	Self-dose	1.19E-03	86	1	49	Self-dose
Pituitary gland	2.45E-03	67	0	28	Brain	1.90E-03	97	1	44	Brain
Spinal cord	3.12E-03	82	2	20	Self-dose	3.26E-03	83	3	22	Self-dose
Ureters	7.53E-03	92	0	17	SI Cont.	9.95E-03	92	0	20	R Colon Cont.
Adipose	3.12E-03	81	0	35	Self-dose	3.07E-03	78	0	46	Self-dose
Trachea	3.40E-03	82	26	—		3.91E-03	81	27	—	
Bronchi	3.56E-03	82	1	23	Lungs	3.44E-03	84	1	27	Lungs
Gall Bladder contents	9.57E-03	94	0	40	R Colon Cont.	6.13E-03	90	0	14	Self-dose
Cartilage	3.59E-03	86	2	15	Self-dose	3.70E-03	84	1	19	Self-dose
Heart contents	4.49E-03	88	1	22	Self-dose	4.23E-03	85	1	27	Self-dose
Remainder tissues	5.54E-03	90	1	—		6.04E-03	89	1	—	
**Effective dose (mSv/MBq)**	**1.01E-02**					**1.13E-02**				

The effective dose is also listed in the end of table.

To make a comparison between the ICRP reference voxel phantom (RVP) and the MIRD stylized phantom, two sets of ratios of the effective dose from RVP to MIRD phantom are illustrated in Fig. 10. This comparison was made to evaluate the differences between effective doses for the two classes of computational phantoms.

The ICRP first introduced the protection quantity, ‘effective dose equivalent’, in *ICRP* *Publication 26*, and then updated this concept in *Publications 60* and *103* with the quantity ‘effective dose’. For example, tissue-weighting factors (*w*_*T*_) are different in the methods proposed by *ICRP Publications 26*, *60* and *103*. In this study, ‘effective dose’ was obtained according to method introduced in *ICRP Publication 103* [8], but *ICRP Publication 53* has reported ‘effective dose equivalent’ for administration of radiopharmaceuticals for the MIRD phantom.

The ‘effective dose’ calculated in this study was compared with the ‘effective dose equivalent’ exactly reported in *ICRP Publication 53* [7] and also with the that for the MIRD phantom calculated using new tissue weighting factors. The latter value was calculated using the organ doses reported in *ICRP Publication 53*.

We have shown that the ratios for thyroid uptake of 5–55% are higher than unity, while in the case of blocked thyroid and technetium incorporation the ratios are less than unity. In addition, the latter set of ratios from RVP to MIRD phantom is overall closer to unity than the former one.

## DISCUSSION

### Iodine-labeled radiopharmaceuticals

#### Variations due to different uptakes

As the thyroid uptake varies from 0–55%, the thyroid gland takes up more and more radioiodine; hence, the absorbed dose to the thyroid is increasing. This occurs because the major contribution of absorbed dose to the thyroid is self-dose. For example, in the case of the male phantom, ∼ 100% and ∼ 99% of the absorbed dose is, respectively, due to ^131^I and ^123^I distributed within the thyroid gland itself. This rising trend is observed for some other organs close to the thyroid gland. For example, the major part of the dose to the esophagus, trachea and salivary glands are due to sources in the thyroid. The contributions listed in Tables 1 and 2 correspond to a normal uptake of 15%, but one can generalize from this to the full range of uptakes. This can be understood by the fact that the dose due to sources in the thyroid goes up as thyroid uptake increases; thus, the thyroid remains the major source irradiating these organs. The esophagus, e.g., has the thyroid as the major contributing source (97% for incorporation of ^131^I, and 79% and 81% for incorporation of ^123^I in male and female model, respectively).

Conversely, a decreasing trend can be observed for organs exposed mainly to sources in the urinary bladder contents. The cumulated activity of the urine reduces with respect to thyroid uptake. When thyroid is blocked or takes less radioiodine, then organs close to urinary bladder acquire a larger amount of dose. Undoubtedly, if the thyroid does not pick up radioiodine it is excreted rapidly by the urinary system. As shown in Fig. 3, voxel doses (mGy/MBq) from photons in the case of the blocked thyroid are higher near the urinary bladder; there is a tendency towards higher values around the thyroid when thyroid uptake is normal.

The absorbed dose to organs that are irradiated mostly from the stomach or small intestine remains relatively constant with increasing thyroid uptake. From this one can assume that uptake of the stomach and small intestine does not vary whether the thyroid takes up more or less iodine.

Moreover, it is assumed in biokinetic data that uptake by the remaining tissues is a constant value. Hence, it would be expected that the absorbed dose to organs whose major dose is from self-irradiation would not change considerably. This can be observed for the testes in the male phantom.

#### Comparison between the two genders

As indicated earlier, target organs in the female phantom generally receive a greater absorbed dose than those in the male phantom, because female phantoms generally have smaller body sizes, i.e. smaller interorgan distances and organ masses.

Indeed, absorbed dose to main source organs such as the thyroid, stomach and small intestine is due to self-irradiation with electrons. In the case of self-irradiation, the influence of organ mass is dominant. Smaller mass corresponds to higher absorbed dose if identical activity has been incorporated to the phantom. For other organs, the distances between main source organs and target organs play an important role in the case of cross-irradiation. A female phantom with shorter body height does have smaller distances between organs and thus receives a larger dose to organs than in the male phantom.

Apparently, radioactive iodine mainly accumulates in thyroid, and that organ has the largest absorbed dose from self-irradiation. One expects to observe the second greatest absorbed dose in the closest organ to the thyroid. The trachea and the lymphatic nodes of the thoracic airways (LN-Th) are both adjacent to the thyroid gland. The second greatest absorbed dose is the trachea in the male and the LN-Th in the female phantom. This observed difference can be explained by CLDs. The CLDs estimated for the thyroid–trachea and thyroid–LN-Th organ pairs are shown in Fig. 4. As indicated in this figure, the CLDs of these two pairs have similar shapes for the female phantom, but the CLD between the thyroid and the trachea is shifted toward smaller values compared with the CLD between the thyroid and the LN-Th in the male phantom. Thus, the energy deposited in the trachea is greater than in the LN-Th in the male phantom.

#### Comparison between photons and electrons

Electrons as charged particles are considered weakly penetrating radiation. Thus electrons emitted within a source organ deposit a significant fraction of energy inside the same organ. Photons, however, can escape from the source organ and penetrate deep inside the body due to their energy. The photon contributions to the total dose as tabulated in Tables 1 and 2 for main source organs, therefore, are small; while for remaining organs the contributions are considerably large. Figure 5 also shows the dose distribution for electrons and photons in a coronal view of the female phantom. This figure indicates that photons contribute a significant dose deep into surrounding tissues while electrons deposit their energy locally in the source region.

#### Comparison between ^*131*^*I and*^*123*^*I*

Absorbed doses to all organs are greater for incorporation of ^131^I than for that of ^123^I. The estimated effective dose for administration of ^131^I is also about two orders of magnitude (100 times) greater than that of ^123^I. This is due to the fact that ^123^Iodine decays by electron capture, and the subsequent radiations have lower energies. Additionally, the physical half-life of ^131^I is 8.02 days while it is 13.27 h for ^123^I. Thus, the effective half-life of the former radionuclide is larger. This results in a greater value of time-integrated activity for source organs, i.e. a longer residence time for ^131^I. The same initial activity for both radionuclides, together with the longer half-life of ^131^I yields higher doses estimated for target organs.

As shown in Tables 1 and 2, the contribution of organ doses due to the thyroid source is higher for ^131^I than for ^123^I. This is another consequence of the greater cumulated activity for the thyroid particularly as the most important source organ.

So, the lower organ doses and effective dose of ^123^I should be considered, particularly for diagnostic procedures, as demonstrated earlier. However, the increased cost of a whole-body scanning with ^123^I must also be considered, because this tracer is not available in many countries.

### Pertechnetate

Cumulated activity of source organs is greater for intravenous than oral administration of pertechnetate. This can be understood from the fact that ^99m^Tc is distributed directly to body organs when the compound is incorporated intravenously. In the case of oral intake, time-integrated activity of the stomach is higher and that of other organs is lower. It is shown in Fig. 9 that the stomach wall and contents have a hotter appearance when ^99m^Tc is given orally. It should be emphasized that the energy deposited in the stomach contents is not an important factor in internal dosimetry. Indeed, only organ doses are important and taken into account for estimation of the effective dose. This figure also shows that adjacent regions of the urinary bladder are more highly irradiated when a blocking agent has been given.

Furthermore, the absorbed doses assessed for the thyroid, stomach, and colon are comparable. It should be noted that technetium does not accumulate mainly in the thyroid gland, as does iodine, so the absorbed dose in the thyroid is not much higher than in other organs.

The effective dose for pertechnetate is estimated to be lower than that for iodine-labeled radiopharmaceuticals. Moreover, the absorbed dose to most organs is greater for intake of iodine-labeled radiopharmaceuticals. In this respect ^99m^Tc has an advantage in nuclear medicine procedures, however it does not provide information needed for subsequent ^131^I treatment about the avidity of the tumor for radioiodine.

### Comparison of effective dose

Considering the second set of effective dose ratios from the RVP to the MIRD phantom is closer to unity, the differences in effective doses are likely a consequence of differences in tissue-weighting factors used to derive each value, rather than variation in the phantoms. The thyroid gland contributes 95–98% of the effective dose for uptake of 5–55%. The gender-averaged absorbed dose to the thyroid in the RVP is greater by ∼ 6–7% than the thyroid dose in the MIRD phantom. This would result in greater than unity effective dose for an uptake range of 5–55%. For incorporation of sodium iodide with a thyroid-blocking agent and ^99m^Tc, all the organs have almost the same contribution in effective dose, and the ratios are less than unity in this case.

## FUNDING

This study was funded by Vice President for Research & Technology of Ferdowsi University of Mashhad. This study was supported by a grant from the Ferdowsi University of Mashhad (no. 20425, 1/3/2012).

## References

[1] Cooper D-S, Doherty G-M (2009). Revised American Thyroid Association management guidelines for patients with thyroid nodules and differentiated thyroid cancer. Thyroid.

[2] Scheff A-M, Lavely W-C, Gelfand M-J (2009). ACR–SNM–SPR practice guideline for the performance of thyroid scintigraphy and uptake measurements.

[3] Sarkar S-D, Becker D-V, Becker BL Thyroid uptake and imaging. Principles and Practice of Endocrinology and Metabolism.

[4] Loevinger R, Berman M-A (1968). A schema for absorbed-dose calculations for biologically distributed radionuclides. J Nucl Med.

[5] Bolch W-E, Eckerman K-F, Sgouros G (2009). MIRD pamphlet No. 21: a generalized schema for radiopharmaceutical dosimetry—standardization of nomenclature. J Nucl Med.

[6] Lamart S, Bouville A, Simon S-L (2011). Comparison of internal dosimetry factors for three classes of adult computational phantoms with emphasis on ^131^I in the thyroid. Phys Med Biol.

[7] ICRP (1987). ICRP Publication 53: Radiation Dose to Patients from Radiopharmaceuticals..

[8] ICRP (2007). ICRP Publication 103: The 2007 Recommendations of the International Commission on Radiological Protection.

[9] ICRP (2003). ICRP Publication 89: Basic Anatomical and Physiological Data for Use in Radiological Protection: Reference Values..

[10] ICRP (2008). ICRP Publication 110: Adult Reference Computational Phantoms.

[11] Waters L-S (1999). MCNPX User's Manual, Version 2.4.0.

[12] ENSDF Decay Data in the MIRD (Medical Internal Radiation Dose) Format for ^131^I http://www.orau.org/ptp/PTP%20Library/library/DOE/bnl/nuclidedata/MIRI.

[13] ICRP (2010). ICRP Publication 116: Conversion Coefficients for Radiological Protection Quantities for External Radiation Exposures.

[14] MIRD (1975). Dose Estimate Report No. 5. Summary of current radiation dose estimates to humans from ^123^I, ^124^I, ^125^I, ^126^I, ^130^I, ^131^I, and ^132^I as sodium iodide. J Nucl Med.

